# Breaking the phase noise–mode purity trade-off in long-loop OEOs via cascaded zero-dispersion recirculating loops

**DOI:** 10.1038/s41377-026-02413-3

**Published:** 2026-09-16

**Authors:** Zhuoran Wang, Santiago Bernal, David V. Plant, Lawrence R. Chen

**Affiliations:** McGill University, Department of Electrical and Computer Engineering, Montreal, QC H3A 0E9 Canada

**Keywords:** Microwave photonics, Fibre optics and optical communications

## Abstract

Optoelectronic oscillators (OEOs) generate microwave signals with ultra-low phase noise by incorporating long optical fiber loops as high-Q energy storage elements. However, long fiber loops reduce the free spectral range, leading to dense oscillation modes and requiring ultra-narrowband filters for single-mode operation, posing a long-standing trade-off between phase noise and mode purity. Here, we present a low-cost and scalable solution that breaks this trade-off. By inserting three cascaded zero-dispersion recirculating loops (ZD-RLs) into an amplified spontaneous emission (ASE)-based OEO, robust single-mode operation is achieved without using ultra-narrowband filters. The longest ZD-RL enhances the Q-factor of the microwave photonic filter (MPF) 4100 times, while the shorter loops increase the peak spacing within the MPF’s passband to enable single-mode operation. This yields a sidemode suppression ratio of 72.7 dB and phase noise of –122 dBc Hz^−1^ at 10 kHz offset at 10 GHz, matching commercial generators. This work shows that even low-cost broadband ASE sources can generate high-quality microwave signals, bridging the gap between laboratory OEO demonstrations and practical signal generators.

## Introduction

Pure microwave signals play a vital role in radar^1,2^, communication^2–6^, and instrumentation^7,8^, serving as local oscillators (LO) for frequency conversion. For example, in a coherent communication system such as 5 G, high-order modulation formats like 256-QAM and 1024-QAM are particularly vulnerable to phase noise, as phase jitter from the LO can distort the signal constellation, causing rotation and blurring that lead to increased bit error rates^9^. Using a low-phase-noise LO is essential for maintaining modulation integrity and communication reliability. As wireless systems evolve toward 6 G, the demand for higher-frequency LOs becomes increasingly critical. However, achieving low-phase noise at high frequencies remains a fundamental bottleneck for conventional electronic signal generators. For example, electronic oscillators, such as quartz oscillators, only exhibit high-quality factors at low frequencies ranging from around 10 to 100 MHz. In order to generate microwave signals in the GHz range, it is typical to multiply the output of these MHz-range high-performance quartz oscillators. However, this multiplication process results in an increase in the phase noise of 20 log_10_*M* dBc Hz^−1^, with *M* being the multiplication factor^10^. As a result, the phase noise quality of the multiplied signals deteriorates as the oscillation frequency rises.

The optoelectronic oscillator (OEO) has emerged as a promising option for generating high-quality microwave signals at high frequencies^11–15^. An OEO is a hybrid system that combines microwave and photonic components with an amplified positive feedback loop to enable microwave oscillation. The low-phase noise performance is enabled by incorporating a long optical fiber in the feedback cavity, which is feasible since the loss of optical fiber is as small as 0.2 dB/km in comparison to that of 360 dB/km (@2 GHz) of a typical microwave coaxial cable^16^. Consequently, OEOs with long-loop length exhibit high Q factor, ensuring low-phase noise signal generation^17^. For example, a record low-phase noise of −163 dBc Hz^−1^ at 6 kHz offset frequency at 10 GHz was demonstrated by using a 16-km-long optical fiber in the OEO loop^18^. However, longer loop length also brings an issue, a smaller free spectral range (FSR). For instance, a cavity length of 16 km yields an FSR of merely 12.5 kHz. Consequently, numerous unwanted side modes appear at integer multiples of the 12.5 kHz offset^18^, which constrains their practical use in precision RF and photonic systems. To ensure single-mode oscillation under such conditions, an ultra-narrow filter with a bandwidth in the kilohertz range is required, posing a significant challenge for both electrical and optical filters. As a result, there is an inherent trade-off between achieving low-phase noise and maintaining pure single-mode operation.

Ever since the invention of OEO^17^, the most widely adopted approach to solve this issue is the multi-loop configuration, which leverages the Vernier effect to effectively increase the FSR^19–21^. A typical configuration employs one long loop to achieve a high Q factor and one or more short loops to broaden the overall FSR. However, the phase noise of the system is influenced by the average phase noise contributions from both the long and short loops^22^. As a result, the presence of short loops inevitably degrades the overall phase noise performance, meaning the fundamental trade-off between low-phase noise and single-mode oscillation still exists. Furthermore, to prevent optical interference between loops, the multi-loop configuration necessitates either multiple photodetectors (PD)^19,20^, polarization multiplexing, which is limited to dual-loop configurations^23,24^, or wavelength multiplexing, which requires multiple laser sources^25,26^. All of these methods significantly increase the system complexity, and none of them has demonstrated a clear path toward achieving spurious-free oscillation, where all unwanted modes are completely suppressed — a standard more readily attainable in purely electrical oscillators. More recently, the concept of parity-time (PT) symmetry has been introduced into OEO architectures to enable single-mode oscillation^27,28^. While promising in theory, this approach requires sophisticated manipulation of gain and loss within a dual-loop cavity, which remains impractical for real-world applications. Moreover, this technique likewise fails to offer a reliable route toward spurious-free oscillation.

In this work, building upon our previous demonstration of a long-loop ASE-based OEO^29^, we transform a noisy multimode oscillation into a clean single-mode oscillation by inserting three low-cost zero-dispersion recirculating loops (ZD-RLs). Importantly, each added ZD-RL does not degrade the phase noise performance but instead further enhances the sidemode suppression ratio (SMSR). This clearly breaks the traditional trade-off between low-phase noise and pure single-mode operation. Owing to the use of an ASE source, which exhibits extremely low coherence, the ZD-RLs are immune to self-interference that would otherwise lead to optical power instability. ASE-based OEOs have been widely studied, where spectral slicing of ASE and fiber dispersion jointly create a multi-tap dispersion-induced microwave photonic filter (DI-MPF) for oscillation selection. However, this ASE-based DI-MPF is generally broadband, leading to insufficient mode selectivity and hence a low SMSR, as exemplified by prior demonstrations^29–31^. Therefore, we incorporate three ZD-RLs into the OEO cavity as auxiliary finite impulse response (FIR) filters with much smaller FSRs, which significantly increase the effective Q factor of the original DI-MPF and thereby enable single-mode oscillation. Three ZD-RLs with lengths of 20 m, 200 m, and 1 km, respectively, are cascaded to suppress the spurs at different frequency regimes away from the center frequency. Zero dispersion property is particularly crucial for RLs to maintain the center frequency and enhance the Q-factor of the DI-MPF. As a result, the proposed OEO generates a microwave signal exhibiting a SMSR of 72.7 dB, which is the highest reported value for ASE-based OEOs with 15 dB improvement to earlier results^32^, and a phase noise of –122 dBc Hz^−1^ at 10 kHz offset, which is already comparable to the performance of a commercial radio frequency (RF) signal generator. Moreover, since the phase noise of the OEO is largely frequency-independent, whereas that of electronic signal generators typically degrades with increasing frequency, the system has strong potential to outperform commercial generators at higher frequencies. The demonstrated frequency tuning range spans from 3 to 11 GHz and can potentially be extended into the millimeter-wave regime by using the electrical-slicing technique^33^, which effectively eliminates the intrinsic baseband and dispersion-induced power fading (DIPF) effect. The frequency drift is 0.72 kHz within 5 minutes without any form of stabilization, surpassing the single-wavelength-based OEOs even when they employ frequency stabilization techniques^34^. Since the traditional trade-off between low-phase noise and single-mode oscillation is effectively removed, our approach provides a clear pathway towards spurious-free operation with low-phase noise by simply cascading more ZD-RLs. The entire system is simple, robust, and cost-effective compared to single-wavelength laser-based OEOs, thereby bridging the gap between laboratory OEO demonstrations and practical signal generators, and holding significant promise for applications in high-precision microwave photonic radar, next-generation wireless communications, and advanced instrumentation systems.

## Results

### Principle

**Fig. 1:**
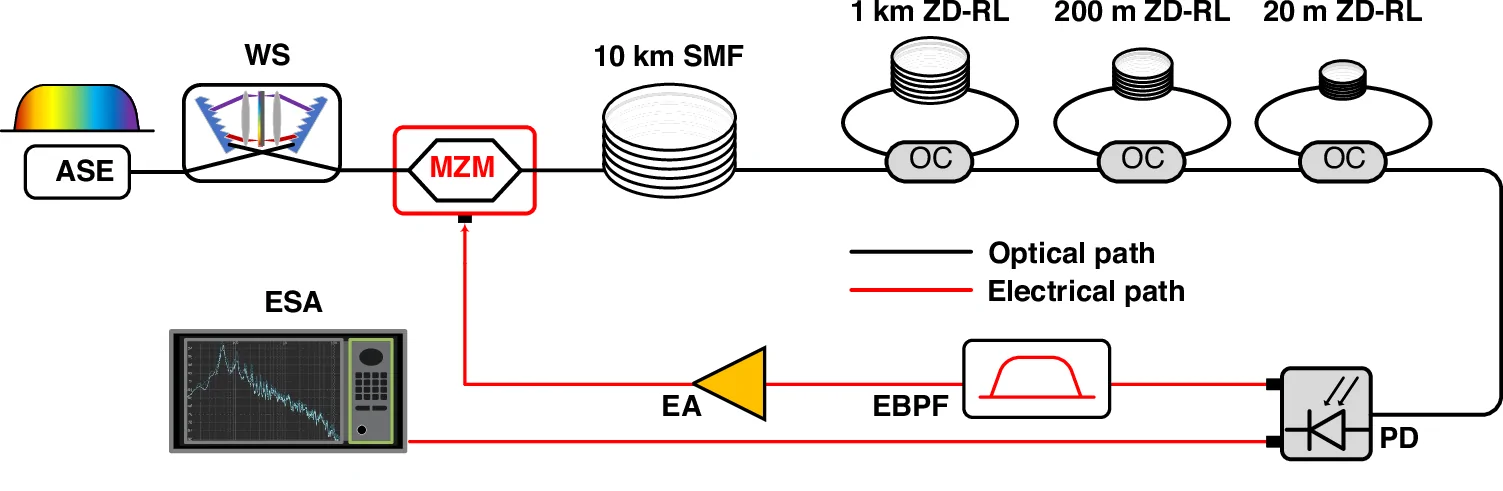
Schematic diagram of our proposed OEO. ASE: amplified spontaneous emission source, which is based on erbium-doped fiber amplifier (EDFA); WS: Waveshaper; MZM: Mach-Zehnder modulator; SMF: single-mode fiber; ZD-RL: zero-dispersion recirculating loop; OC: optical coupler; PD: photodetector; EBPF: electrical bandpass filter; EA: electrical amplifier; ESA: electrical spectrum analyzer

The proposed OEO’s architecture is illustrated in Fig. 1, which is a typical ASE-based OEO^29,30,35^, with the addition of three cascaded ZD-RLs. The ASE source’s spectrum is shaped by a programmable optical filter (e.g., Waveshaper) according to a cosine function, expressed as:

1 $$T\left(\lambda \right)=\frac{1}{2}\left[1+\cos \left(2\pi \frac{\lambda -{\lambda }_{0}}{\varDelta \lambda }\right)\right]$$

where $\varDelta \lambda$ is the slicing period of the optical spectral shaper and ${\lambda }_{0}$ is the center wavelength of the ASE spectrum. Each period in the spectral shape corresponds to a distinct tap. Due to the chromatic dispersion (CD) of the optical fiber, these taps experience different group delays. The delay between adjacent spectral taps is given by:

2 $$\varDelta {\tau }_{\mathrm{disp}}=D\cdot L\cdot \varDelta \lambda$$

where D (ps (km·nm)^-1^) and L are the value of the CD and length of the dispersive medium, respectively. As a result, an MPF is effectively generated within the cavity, with its center frequency located at

3 $${f}_{0}=\frac{1}{\varDelta {\tau }_{\mathrm{disp}}}$$

**Fig. 2:**
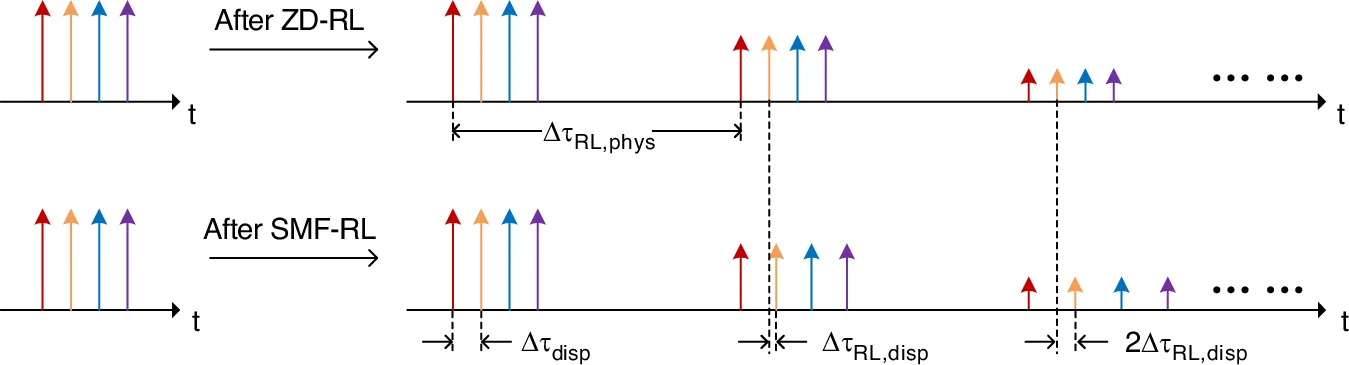
Illustration of tap distributions after propagation through an RL with and without dispersion. For clarity, the model is simplified using discrete taps. Different colors represent different wavelengths

By changing the slicing period, the center frequency of the MPF can be effectively tuned. However, the bandwidth of the resulting MPF is typically on the order of hundreds of megahertz^36^, which is not narrow enough to select a single oscillation mode when a long fiber is used in the OEO cavity, as was the case in our previous work^29^. For example, a 10 km fiber leads to a cavity FSR of only 20 kHz, requiring a filter with kilohertz-level bandwidth. To address this, we introduce optical RLs in the cavity to enhance the Q factor of the MPF. The RL is built by connecting one output port of a 3 dB coupler to one of its input ports with a length of fiber inserted in between. Since an ASE source is used, the issue of self-interference — caused by coherent feedback that would otherwise cause power instability at the output of the RL—is avoided. As illustrated in Fig. 2, each time the signal circulates through the RL, it accumulates:

- A fixed round-trip propagation delay:
  4 $$\varDelta {\tau }_{\mathrm{RL},\mathrm{phys}}=\frac{{L}_{\mathrm{RL}}}{v}$$
  where ${L}_{\mathrm{RL}}$ is the length of the RL, and v is the speed of light in optical fiber
- A wavelength-dependent dispersion delay:

5 $$\varDelta {\tau }_{\mathrm{RL},\mathrm{disp}}={D}_{\mathrm{RL}}\cdot {L}_{\mathrm{RL}}\cdot \varDelta \lambda$$

where ${D}_{\mathrm{RL}}$ is the CD of the fiber inserted in RL. For clarity, we neglect the effect of the dispersion slope. A full derivation that includes dispersion slope, along with a quantitative analysis of its impact on the filter response, is provided in the Supplementary Information, where we show that its influence is negligible under practical conditions.

Hence, for the i-th wavelength tap and the n-th loop circulation, the total delay is:

6 $$\varDelta {\tau }_{i,n}=\left(i-1\right)\cdot \varDelta {\tau }_{\mathrm{disp}}+n\cdot \left(\varDelta {\tau }_{\mathrm{RL},\mathrm{phys}}+\left(i-1\right)\cdot \varDelta {\tau }_{\mathrm{RL},\mathrm{disp}}\right)$$

For clarity, discrete models are used for calculation. The real continuous taps can be regarded as a large number of discrete taps. Each feedback circulation contributes an attenuated replica of the original tap, with an attenuation factor ${\alpha }^{n}$, where α<1 is the loop’s round-trip power attenuation. As a result, the overall RF transfer function of the filter, combining both the FIR taps from the spectrum slicing and the recursive taps from the RL, is given by:

7 $$H\left(f\right)=\mathop{\sum }\limits_{i=1}^{N}\mathop{\sum }\limits_{n=0}^{{N}_{\mathrm{loop}}}{a}_{i}\cdot {\alpha }^{n}\cdot {e}^{-j2\pi f\cdot \varDelta {\tau }_{i,n}}$$

Here, ${a}_{i}$ represents the amplitude of the i-th spectral component determined by the shaping function $T\left(\lambda \right)$, and ${N}_{\mathrm{loop}}$ is the number of loop recirculations considered in the model. Crucially, to maintain the filter performance, the fiber used in the RL must exhibit near-zero dispersion. If single-mode fiber (SMF) is used, the signal experiences additional dispersion delay during each circulation, as shown in Fig. 2. According to Eq. (3), this accumulation induces a progressive shift in the center frequency of the MPF after every loop round-trip. In this case, RLs only introduce additional losses and do not contribute to any *Q*-factor enhancement. To mitigate this issue, a fiber segment with near-zero dispersion is introduced within the RL. This approach effectively suppresses the cumulative dispersion-induced frequency shift, stabilizing the filter’s center frequency and significantly enhancing the *Q*-factor. Detailed simulations comparing SMF-RL and ZD-RL are presented in Supplementary Information.

**Fig. 3:**
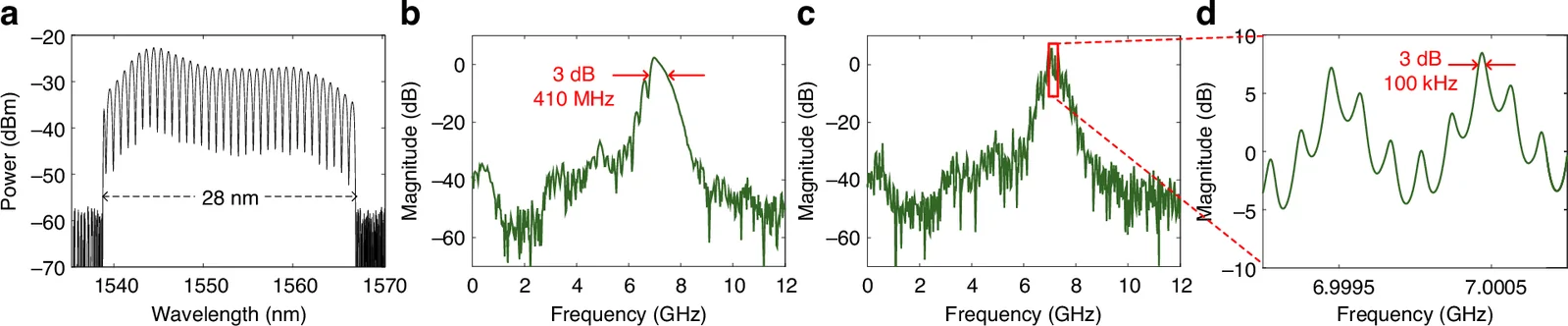
Q-factor enhancement achieved by cascading ZD-RLs. **a** Sliced ASE spectrum measured using an optical spectrum analyzer (OSA) with a resolution bandwidth (RBW) of 0.05 nm. **b** Measured filter response without ZD-RLs, obtained using a vector network analyzer (VNA) with an intermediate-frequency bandwidth (IFBW) of 100 kHz. **c** Measured filter response with three cascaded ZD-RLs. **d** Zoomed-in view of the red boxed region in (**c**), measured with an IFBW of 100 Hz

Experimental measurements are done to prove the *Q*-factor enhancing effect. For example, when the fiber length is 10 km, the ASE bandwidth is 28 nm, and the slicing period is 0.77 nm, as shown in Fig. 3(a), introducing three ZD-RLs of 20 m, 200 m, and 1 km reduces the 3 dB bandwidth from 410 MHz to 100 kHz, as shown in Fig. 3(b-d), indicating a Q-factor enhancement by a factor of 4100. Theoretically, an arbitrarily high Q-factor can be achieved as long as an ultra-long ZD-RL is available. Therefore, in principle, single-mode oscillation can be realized in any long-loop OEO using this approach. Additionally, two FSRs are observed in Fig. 3(d): the smaller one, 200 kHz, is introduced by the 1 km ZD-RL, while the larger one, 1 MHz, corresponds to the 200 m ZD-RL. A third FSR of 10 MHz, originating from the 20 m ZD-RL, also exists but lies outside the frequency range displayed in the figure. These additional FSRs are governed by the round-trip delay of the loop and can be approximated by

8 $${FSR}=\frac{1}{\varDelta {\tau }_{\mathrm{RL},\mathrm{phys}}}$$

In order to expand this RL-induced FSR, we cascaded three ZD-RLs with different lengths. In this configuration, similar to the Vernier effect, the overall Q factor of the composite filter is primarily determined by the ZD-RL with the longest fiber length, whereas the overall FSR is governed by the least common multiple of the individual FSRs. However, unlike multi-loop configurations that utilize the Vernier effect, this approach avoids introducing parallel short loops and thus will not cause phase noise degradation.

### OEO results

**Fig. 4:**
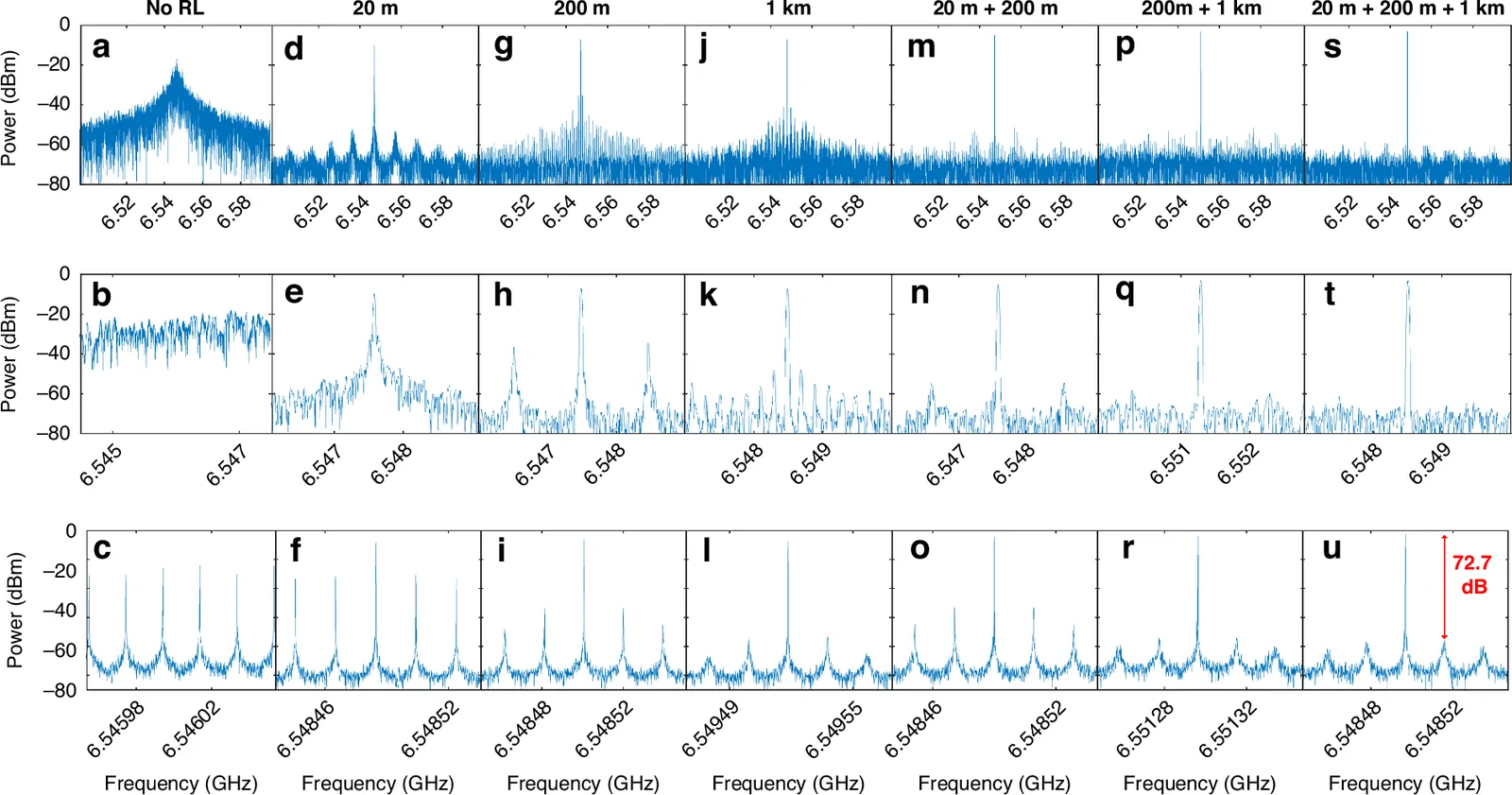
Measured RF spectra under different ZD-RL configurations, obtained using an electrical spectrum analyzer (ESA). The first to third rows correspond to measurements with spans of 100 MHz (**a**, **d**, **g**, **j**, **m**, **p**, **s**), 3 MHz (**b**, **e**, **h**, **k**, **n**, **q**, **t**), and 100 kHz (**c**, **f**, **i**, **l**, **o**, **r**, **u**), respectively. The RBW is set to 20 kHz for the first two rows and 47 Hz for the third row. From left to right, the columns represent the following configurations: no ZD-RL (**a**–**c**); a single 20 m ZD-RL (**d**−**f**); a single 200 m ZD-RL (**g**–**i**); a single 1 km ZD-RL (**j**–**l**); cascaded 20 m and 200 m ZD-RLs (**m**–**o**); cascaded 200 m and 1 km ZD-RLs (**p**–**r**); cascaded 20 m, 200 m, and 1 km ZD-RLs (**s**–**u**)

The proposed OEO was assembled and experimentally tested (see methods). Initially, no ZD-RLs were included in the OEO cavity. The spectrum slicing period was set to 0.8 nm, resulting in an MPF centered at 6.5 GHz. Upon closing the loop, the OEO began to oscillate. The measured RF spectra are shown in Fig. 4(a–c), corresponding to measurement spans of 100 MHz, 3 MHz, and 100 kHz, respectively. As shown, without ZD-RLs, the OEO operates in a noisy multimode state with an FSR of approximately 20 kHz. Next, we inserted a 20 m ZD-RL into the cavity. The OEO transitioned to single-mode oscillation but exhibited significant sidemode noise. Replacing the 20 m ZD-RL with a 200 m one improved the SMSR; however, unwanted modes appeared periodically at integer multiples of 1 MHz from the center frequency, corresponding to the FSR of the 200 m loop. We then increased the ZD-RL length to 1 km, resulting in higher SMSR. Nonetheless, unwanted modes were observed at 200 kHz intervals, again due to the loop’s FSR. Next, two ZD-RLs with lengths of 20 m and 200 m were cascaded. This configuration achieved an SMSR comparable to that of the 200 m ZD-RL alone, while the spurious peaks at integer multiples of 1 MHz offset were suppressed by approximately 20 dB. We then cascaded the 200 m and 1 km ZD-RLs. In this case, the spectrum appears noisier in the 100 MHz span due to the absence of the 20 m ZD-RL, but it becomes cleaner in the 3 MHz and 100 kHz spans owing to the presence of the 1 km ZD-RL. Finally, we cascaded all three ZD-RLs (20 m, 200 m, and 1 km), and got an SMSR as high as 72.7 dB — representing the highest reported value for ASE-based OEOs. Moreover, no unwanted peaks were observed over a wider frequency range, including the 200 kHz and 1 MHz offsets that were previously present when using the 1 km or 200 m ZD-RL alone. This demonstrates a clear transition from noisy multi-mode oscillation, as shown in Fig. 4(a–c), to pure single-mode oscillation, as shown in Fig. 4(s–u), by simply adding three low-cost ZD-RLs into the cavity, demonstrating the strong side-mode suppression capability of our approach.

**Fig. 5:**
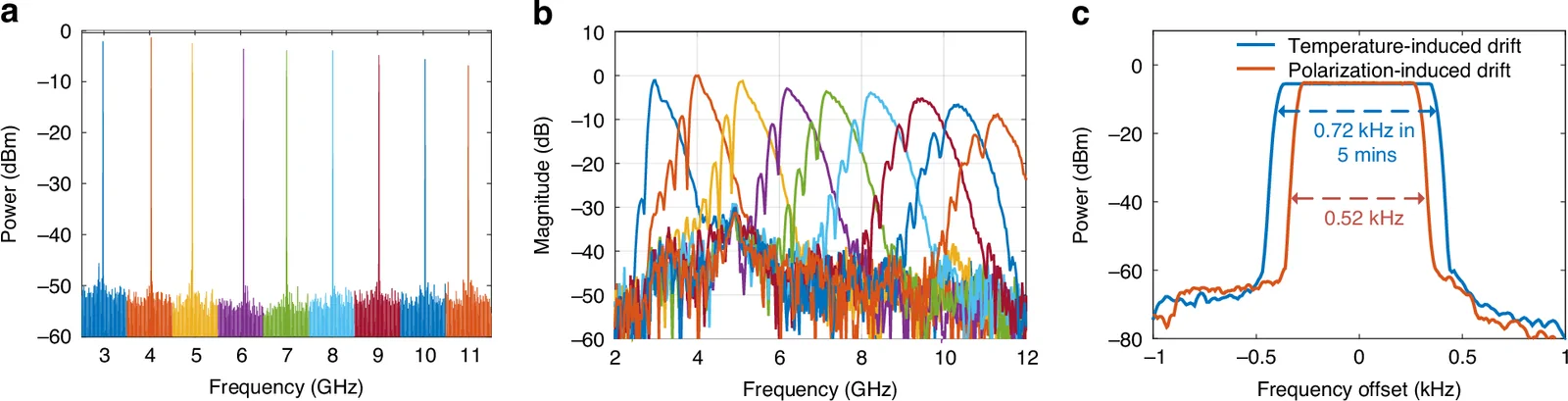
Frequency tunability and stability demonstration. **a** Measured frequency tunability of the RF output using an ESA with RBW of 160 kHz. **b** Measured frequency tunability of the corresponding MPF using a VNA with IFBW of 100 kHz. **c** Measured frequency stability

Then, we demonstrated the frequency tunability. According to Eq. (3), as the slicing period is adjusted from 1.8 nm to 0.5 nm, the oscillation frequency is tuned from 3 GHz to 11 GHz, as shown in Fig. 5(a). The frequency tuning range is limited by three factors. The first is the baseband response of the MPF. Because all optical taps are positive, a strong baseband component is always present, which prevents oscillation from occurring at higher frequencies. To remove the baseband, we have to use an electrical bandpass filter covering 3 GHz to 10 GHz, which limits the upper frequency boundary. The second is the DIPF effect, which arises from interference between the optical carrier and modulation sidebands after transmission through a dispersive medium. This effect introduces a null in the frequency response around 19 GHz when 10 km SMF is used, setting an upper limit on the oscillation frequency. Those two problems can be solved simultaneously by employing the electrical-slicing approach^33^ (see discussion). The third factor is third-order dispersion (TOD), which causes passband amplitude degradation at higher frequencies^36^. Figure 5(b) shows the measured filter responses corresponding to the oscillation frequencies in Fig. 5(a). The filter amplitude decreases by 8.8 dB as the frequency increases from 4 GHz to 11 GHz, due to the combined effects of DIPF and TOD, and this results in a 5 dB reduction in RF output power. However, this limitation can be mitigated either by applying our recently proposed chirp-like slicing technique using the WS^36^, phase pre-compensation technique^37,38^, or by using dispersion-profiled fiber with near-zero dispersion slope^39^, which eliminates the need for the WS and simplifies the system.

We also measured the frequency stability of the generated signal. As shown in Fig. 5(c), the frequency drift within 5 minutes is around 0.72 kHz at 6.5 GHz, primarily due to ambient temperature fluctuations in the lab. Notably, the system was placed in a room-temperature lab without any form of vibration or sound isolation, demonstrating the intrinsic robustness of the proposed OEO. Such stability is challenging to achieve in OEOs employing MPFs based on single-wavelength lasers, which are highly susceptible to optical wavelength or resonance drift. For example, a micro-ring resonator-based OEO exhibits a drift up to 40 kHz in an hour, even with the aid of a frequency offset circuit^34^. This comparison highlights the reliability of our ASE-based approach, which eliminates the need for active stabilization while maintaining excellent frequency stability. Furthermore, to verify the polarization insensitivity of the system, we inserted a polarization controller (PC) after the modulator and arbitrarily rotated all three paddles. The output frequency changed by only ~0.52 kHz, as also plotted in Fig. 5(c), confirming that the OEO performance is effectively insensitive to input polarization state.

**Fig. 6:**
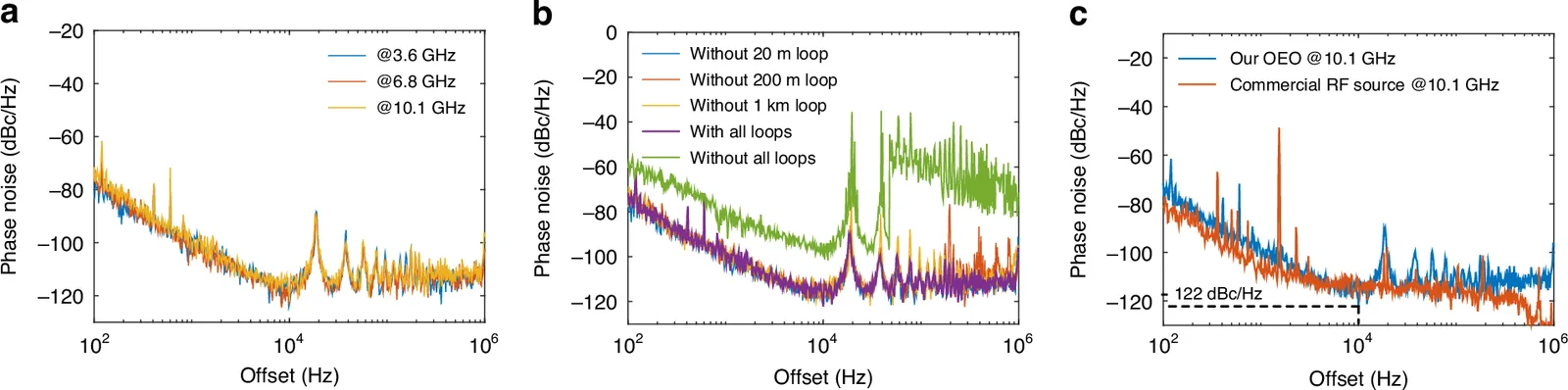
Measured phase noise performance. **a** phase noise performance at different frequencies; (**b**) phase noise performance under different ZD-RLs configurations; (**c**) phase noise comparison between our OEO and a commercial RF source

Finally, we evaluated the phase noise performance. Figure 6(a) shows the phase noise performance at different frequencies, giving evidence that phase noise does not degrade with the increase of frequency. In order to verify whether the phase noise degrades with adding ZD-RL, we measured the phase noise under various ZD-RL configurations. As shown in Fig. 6(b), adding ZD-RLs does not degrade phase noise performance, but rather helps suppress side modes, verifying that the traditional trade-off between high SMSR and low-phase noise has been eliminated. This gives the possibility to achieve spurious-free oscillation by simply adding more ZD-RLs. We also compared our generated signal with a commercial RF source. By adjusting the slicing period to 0.55 nm, we generated a 10.1 GHz RF signal, with phase noise of −122 dBc Hz^−1^ at 10 kHz offset. This level of phase noise performance is already comparable to a commercial RF signal generator (Anritsu, MG3692B), as shown in Fig. 6(c). Further phase noise reduction is possible by employing a lower-noise electrical amplifier (EA), since the EA (SHF, 810) used in our experiment has a relatively high noise figure of 6 dB. Additionally, optimizing the link loss could yield improvements, since the lab setup introduces considerable unnecessary insertion loss due to the use of multiple connectors. Given that commercial RF signal generators typically suffer from phase noise degradation as frequency increases, an issue not observed in our system, our method holds significant potential to outperform them at higher frequencies once the tuning range is expanded.

## Discussion

A comprehensive performance comparison between the proposed OEO and other previously reported MPF-based OEOs is presented in Table 1. OEOs employing electrical bandpass filters are excluded from this comparison, as their use contradicts the original motivation for adopting microwave photonics and inherently limits frequency tunability. As shown in the table, our design employs the longest fiber length among the compared works, resulting in the smallest FSR. Nevertheless, it achieves the highest SMSR, demonstrating the unprecedented side-mode suppression capability enabled by the cascaded ZD-RLs. The SMSR of 72.7 dB represents the highest reported value for ASE-based OEOs, marking a 15 dB improvement over previously reported work^32^. Notably, this enhancement is achieved despite our use of a significantly longer fiber length (10 km compared to 0.5 km in the earlier work^32^), which would typically make side-mode suppression more challenging, suggesting that the actual improvement is even more substantial. While the phase noise performance does not surpass that of dual-loop SBS (stimulated Brillouin scattering)-based OEOs, it remains acceptably low given the simplicity of our architecture, which avoids multiple parallel cavities and the associated need for costly stabilized tunable lasers. Note that the measured frequency drift in this work is more than three times larger than in our previous result. This increase is not caused by the inclusion of the RLs, but is instead due to measurement-to-measurement variation driven by ambient temperature fluctuations. A detailed analysis is provided in the Supplementary Information. Although the current implementation involves a programmable optical filter (WS), which increases system cost, it serves solely as a spectrum slicer without doing any spectral apodization. Therefore, the WS can be readily replaced by a cost-effective Mach-Zehnder Interferometer (MZI), making the overall system economically viable. Moreover, our OEO demonstrates excellent frequency stability without the need for any frequency stabilization techniques, unlike SBS-based OEOs that require multiple commercially stabilized tunable lasers, making our approach more practical for real-world applications.

**Table 1:** Performance Comparison of MPF-based OEOs

Light source	Approach	OEO length (km)	SMSR (dB)	Phase noise (dBc Hz^−1^)	Frequency tunability (GHz)	Frequency stability
SW^42^	PS-FBG	0.5	–	−102	3–28	–
SW^15^	MRR	0.025	50	−102	20–35	28 kHz in 8 m
SW^43^	MRR	0.04	50	−88	14–25	250 kHz in 5 m
SW^34^	MRR + FOC	0.12	37	−98	4–12	40 kHz in 1 h
SW^44^	SBS	1	–	−113	0–40	–
SW^45^	SBS + DL	2/4	35	−100	0–60	–
SW^46^	SBS + DL	1.1/1.2	–	−128	7–40	5.7 kHz in 16 m
ASE^30^	DI-MPF	6.9	–	−120	4–9.7	–
ASE^29^	DI-MPF	10	Multi	−100	3–11	0.2 kHz in 5 m
ASE^31^	DI-MPF	3.5	–	−107	1–12	–
ASE^32^	DI-MPF + DL	0.5/0.7	57	−97	4–13	11.6 kHz in 1 h
ASE (this work)	DI-MPF + ZD-RLs	10	72.7	−122	3–11	0.72 kHz in 5 m

“-“ represents not reported, *Multi* multimode oscillation, *SW* single-wavelength, *SBS* stimulated Brillouin scattering, *PS-FBG* phase-shifted fiber Bragg grating, *MRR* micro-ring resonator, *FOC* frequency offset circuit, *DL* dual loop, *DI-MPF* dispersion-induced microwave photonic filter

**Fig. 7:**
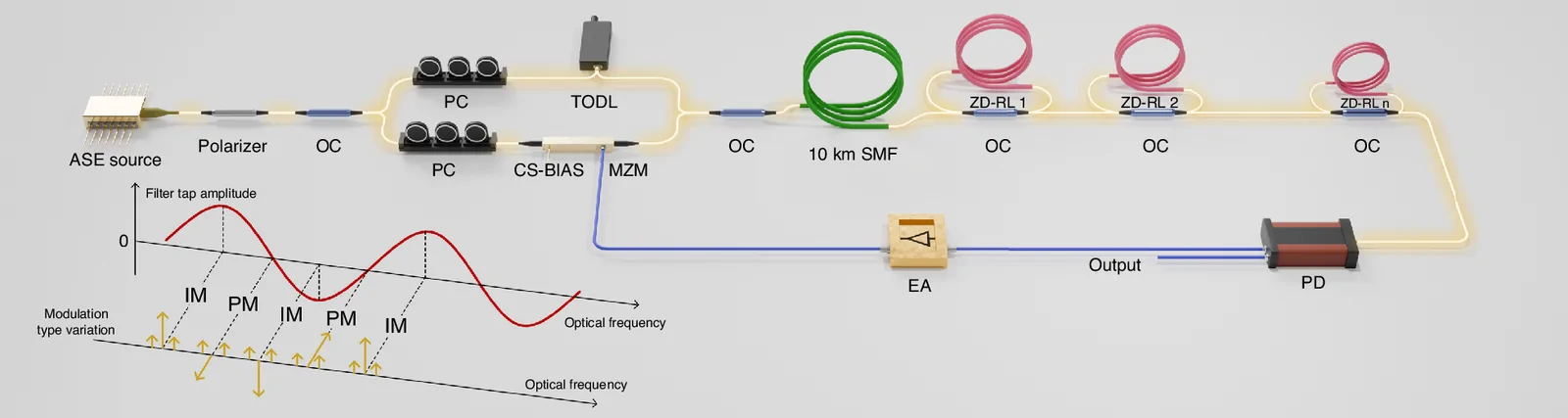
Schematic diagram of envisioned OEO. ASE: amplified spontaneous emission source (butterfly diode package, e.g., SLD; not the source used in our experiment); PC: polarization controller; CS-BIAS: carrier-suppression bias; TODL: tunable optical delay line; MZM: Mach-Zehnder modulator; SMF: single-mode fiber; ZD-RL: zero-dispersion recirculating loop; OC: optical coupler; PD: photodetector; EA: electrical amplifier; ESA: electrical spectrum analyzer

The most encouraging aspect is that the limitations of this configuration have not yet been reached, primarily due to the limited number of usable ZD-RLs available in our laboratory. Since the addition of ZD-RLs does not degrade the phase noise but instead improves the SMSR, more ZD-RLs can be incorporated as long as sufficient gain is maintained to sustain oscillation. Consequently, it is straightforward to anticipate a further improvement in SMSR with the inclusion of additional ZD-RLs. Therefore, we proposed an envisioned OEO architecture with more ZD-RLs cascaded, as shown in Fig. 7, which outlines a clear and practical approach for generating a spurious-free signal, a standard that is more readily attainable in purely electrical oscillators.

Furthermore, unlike our current configuration, the envisioned architecture does not rely on optical slicing. Instead, it employs electrical slicing, realized by periodically alternating the modulation state between intensity modulation and phase modulation^33,40^. This variation arises from the dispersion difference between the upper and lower arms of the MZI structure. By biasing the Mach-Zehnder modulator (MZM) at the carrier-suppression point, the baseband of the MPF is removed, as electrical slicing—unlike optical slicing—naturally allows for negative tap coefficients. Furthermore, the DIPF effect, caused by dispersion-induced modulation type evolution (DIME), is also mitigated, as in this case, DIME only introduces a phase shift in the electrical-slicing signal, without affecting its amplitude. Consequently, the filter’s amplitude remains stable across a wide frequency range. For completeness, this architecture does introduce two practical issues. First, the MZI is environmentally sensitive, particularly to temperature fluctuations, which can compromise the frequency stability. This can be addressed through active feedback control to stabilize the interferometer bias. Second, the MZI requires polarization management. A polarization controller in the upper arm is needed to align its polarization state with that of the lower arm in order to maximize the RF beat signal at the photodiode. This requirement can be relaxed by implementing polarization-maintaining fiber within the MZI structure. Nevertheless, a single-bandpass MPF operating in the W-band has already been experimentally demonstrated using this electrical-slicing concept^37,38^. Considering that benefit, which is far more valuable than the inconvenience of the two above engineering constraints, we believe that combining the proposed cascaded ZD-RL technique with electrical-slicing offers a promising solution to the current limitation in frequency tuning range, and could enable spurious-free oscillation in the millimeter-wave regime with ultra-low-phase noise, thereby truly outperforming conventional signal generators based on pure electronics.

It is worth noting that in the present work, we focused on addressing the fundamental question, “how can we significantly enhance the SMSR without compromising the phase noise performance?” Our findings in this paper show that using cascaded ZD-RLs allows us to overcome this long-standing SMSR vs phase noise trade-off. Future research can explore further the flexibility of ASE-based OEOs, such as investigating parameters such as filter bandwidth, the number of channels, and the passband shape that can all be reconfigured, as well as applications and extensions. One such extension is our recent work where we use the idea of cascaded ZD-RLs developed in this work in conjunction with (1) the hybrid spectrum slicing technique for generating a dual-band MPF and (2) mutual injection locking to overcome mode competition to demonstrate an independently tunable dual-frequency OEO^41^. Specifically, in that work, we exploit the cascaded ZD-RLs as an enabling technology for achieving single-mode oscillation at both frequency tones of the dual-frequency OEO; without the ZD-RLs, severe mode noise would arise, leading to degraded phase-noise performance. These results highlight the significance of the work reported here and the potential of ASE-based OEOs as flexible platforms for high-performance microwave signal generation.

In summary, we have demonstrated a novel single-mode OEO architecture that leverages an ASE source and cascaded ZD-RLs to achieve high-purity microwave signal generation. By simply inserting three ZD-RLs with varying lengths, the system significantly enhances the Q-factor of the MPF while expanding the MPF’s FSR, enabling stable single-mode oscillation without the need for ultra-narrowband filters. Importantly, each added ZD-RL does not degrade the phase noise performance but instead further enhances the SMSR, breaking the traditional trade-off between low-phase noise and pure single-mode operation. Experimentally, the proposed OEO generates a microwave signal with a sidemode suppression ratio of 72.7 dB, representing the highest reported value for ASE-based OEOs, and phase noise of −122 dBc Hz^−1^ at 10 kHz offset at 10 GHz, performance that rivals commercial signal generators. The frequency tuning range spans from 3 to 11 GHz and can potentially be extended to the millimeter-wave regime, with a frequency drift of only 0.72 kHz over five minutes without any form of stabilization. Since the trade-off between low-phase noise and pure single-mode operation is eliminated, our approach provides a clear pathway towards spurious-free operation by simply cascading more ZD-RLs. With its robustness, simplicity, scalability, and cost-effectiveness, this work moves a step closer to realizing practical high-quality microwave photonic signal-generating sources.

## Materials and methods

The schematic diagram of the proposed single-mode OEO is illustrated in Fig. 1. The system begins with an ASE source, which is a high-power EDFA (erbium-doped fiber amplifier), delivering broadband optical noise centered at 1553 nm, with a total spectral width of 28 nm and output power of 25 dBm (the exact ASE bandwidth chosen has little impact on the overall OEO performance, as discussed in the Supplementary Information). This broadband light is first directed into a programmable optical filter (Finisar Waveshaper), which only performs optical spectrum slicing without any spectrum apodization. Therefore, the WS can be replaced by a simple MZI structure in the future. The spectrally sliced ASE light is then modulated by a MZM, which is biased at the quadrature point. The modulated optical signal travels through a 10 km SMF to introduce delay. It then passes through three ZD-RLs with lengths of 1 km, 200 m, and 20 m, which enhance mode selectivity and enable single-mode oscillation. To achieve near-zero dispersion, the 1 km and 200 m ZD-RLs are carefully engineered using appropriate length combinations of SMF and dispersion-compensated fiber (DCF), resulting in measured CD values of -0.69 ps (km·nm)^-1^ and -0.04 ps (km·nm)^-1^, respectively. Because the dispersion slope has negligible impact on the filter response (see Supplementary Information), a standard dispersion-shifted fiber (DSF), whose dispersion is engineered to be near zero at 1550 nm, is also suitable for use in this architecture. The 20 m ZD-RL, by contrast, consists solely of a 20 m segment of SMF, as its short length makes dispersion negligible. The measured insertion losses (IL) of the 20 m, 200 m, and 1 km ZD-RLs are 0.8 dB, 1.2 dB, and 3.0 dB, respectively. The ZD-RL IL was measured with an optical power meter as an end-to-end power difference between the laser output and the ZD-RL output, including one connector/adapter interface. The connector/adapter loss could be reduced by replacing the connectorized interfaces with direct splicing, and the overall ZD-RL insertion loss could be further lowered through improved SMF–DCF splicing, which helps further increase the SMSR. The optical signal is then converted into the electrical domain by a PD with two output ports. One port is used for signal generation: the output is first passed through an electrical bandpass filter from 3 GHz to 10 GHz to remove the baseband. The filtered signal is then amplified by an EA to a suitable drive level before being fed back into the MZM, completing the feedback loop required for sustained oscillation. The second port of the PD is connected to an electrical spectrum analyzer (ESA) for real-time observation and analysis of the generated RF signal.

## Supplementary information

Supplementary Information

## Data Availability

Data underlying the results presented in this paper may be obtained from the authors upon reasonable request.
