# Supplementary material for: Breaking the phase noise–mode purity trade-off in long-loop OEOs via cascaded zero-dispersion recirculating loops
Source: Light Sci Appl. 2026 Sep 16;15:379. doi: 10.1038/s41377-026-02413-3 (PMC13578445; doi:10.1038/s41377-026-02413-3)
Supplement: Supplementary file 1 [file 41377_2026_2413_MOESM1_ESM.docx]

**Supplementary Information for Breaking the Phase Noise–Mode Purity Trade-Off in Long-Loop OEOs via Cascaded Zero-Dispersion Recirculating Loops**

Zhuoran Wang, Santiago Bernal, David V. Plant, Lawrence R. Chen

McGill University, Department of Electrical and Computer Engineering, Montreal, QC H3A 0E9, Canada

**Supplementary Note 1 | Numerical comparison between SMF-RL and ZD-RL**

To investigate the impact of CD in the RL, we simulate the filter response in MATLAB according to Eq. 7, with the simulation parameters summarized in Table 1. As a reference, Supplementary Fig. S1(a) shows the amplitude response in the absence of any RLs. When a 1 km SMF with a CD of 18.5 ps (km·nm)^-1^ is inserted into the RL, the filter exhibits a 3 dB amplitude reduction due to the insertion loss of the RL, and negligible enhancement in the Q-factor of the highest passband, as shown in Supplementary Fig. S1(b-c). However, when the SMF is replaced with a zero-dispersion fiber, dispersion-induced degradation is effectively mitigated; consequently, the filter's amplitude remains unchanged, and the Q-factor is significantly improved, as illustrated in Supplementary Fig. S1(d-e). Moreover, a new periodic spectral feature is observed in the frequency response, as shown in Supplementary Fig. S1(e) corresponding to the FSR introduced by the 1 km RL.

Table 1. List of the simulation parameters

| **Parameters** | **Value** |
| --- | --- |
| SMF length | 10 km |
| CD | 18.5 ps (km·nm)^-1^ @ 1550 nm |
| Slicing period | 0.77 nm |
| ASE bandwidth | 28 nm |
| RL length | 1 km |
| RL CD | 18.5 or 0 ps (km·nm)^-1^ @ 1550 nm |
| Attenuation factor | 0.5 |

Fig. S1. Simulated MPF response. (a) MPF response without any RLs. (b) MPF response with a 1 km SMF-RL. (c) Zoomed-in plot of the rectangular area in (b). (d) MPF response with a 1 km ZD-RL. (e) Zoomed-in plot of the rectangular area in (d).

**Supplementary Note 2 | Investigation of the Effect of RL’s Dispersion Slope**

In the main text, the recirculating loop (RL) in the proposed optoelectronic oscillator is modeled as a dispersion element that provides wavelength-dependent group delay. For simplicity, the RL’s dispersion was initially treated as constant across the ASE slicing bandwidth. Under this assumption, the dispersion-induced differential group delay between two adjacent optical taps separated by a fixed wavelength spacing $\Delta\lambda$ can be written as

$$\Delta\tau_{\text{RL,disp}}=D_{\text{RL}}\cdot L_{\text{RL}}\cdot\Delta\lambda$$

(S1)

where $D_{\mathrm{RL}}$ is the fiber dispersion at the center wavelength $\lambda_{0}$, $L_{\mathrm{RL}}$ is the loop length. This assumes the dispersion $D_{\mathrm{RL}}$ is constant across the optical spectrum. However, in practical implementations, such as when using dispersion-shifted fiber (DSF), the dispersion is not strictly constant across the slicing bandwidth. Although the dispersion at $\lambda_{0}\approx1550\text{ }$can be engineered to be close to zero, it varies with wavelength. This variation is commonly characterized by the dispersion slope $S_{\mathrm{RL}}$ (units: ps (km·nm^2^)^-1^). As a result, the incremental group delay contribution from the RL is no longer constant across the spectrum and becomes wavelength dependent. In this section, we derive the corrected expression for the RL’s dispersion-induced delay when the dispersion slope is taken into account.

1. **Derivation of RL’s dispersion-induced delay with considering dispersion slope**

We model the wavelength-dependent dispersion of the RL fiber as a first-order expansion around a reference wavelength $\lambda_{0}$:

$$D_{\text{RL}}\left( \lambda\right)=D_{\text{RL},0}+S_{\text{RL}}\left( \lambda-\lambda_{0} \right)$$

(S2)

where $D_{\mathrm{RL},0}$ is the dispersion at $\lambda_{0}$, $S_{\mathrm{RL}}$ is the dispersion slope of the RL fiber, $\lambda$ is the optical wavelength. We consider an ASE source that is spectrally sliced into discrete taps. Let $\lambda_{i}$ denote the wavelength of the $i$-th tap, and let $\Delta\lambda$ denote the fixed wavelength spacing between adjacent taps:

$$\lambda_{i+1}=\lambda_{i}+\Delta\lambda$$

(S3)

Here, $\Delta\lambda$ is determined by the slicing configuration (e.g., the spacing between adjacent notches of the optical filter), and is therefore a design constant. Importantly, $\Delta\lambda$ in Eq. (S3) is not a small deviation variable; it is the fixed period of the wavelength sampling.

We are interested in the incremental group delay difference between tap $i+1$and tap $i$ that arises from the RL dispersion. Physically, this quantity tells us how much additional round-trip delay the RL introduces for one wavelength slice compared to the next. This incremental delay for one circulation of the RL is given by integrating the wavelength-dependent dispersion between $\lambda_{i}$ and $\lambda_{i+1}$:

$$\Delta\tau_{\text{RL,disp}}\left( \lambda_{i} \right)=L_{RL}\int_{\lambda_{i}}^{\lambda_{i+1}} D_{\text{RL}}\left( \lambda\right) \text{d}\lambda$$

(S4)

Substituting Eq. (S2) into Eq. (S4) yields

$$\Delta\tau_{\text{RL,disp}}\left( \lambda_{i} \right)=L_{\mathrm{RL}}\int_{\lambda_{i}}^{\lambda_{i+1}} D_{\text{RL},0}+S_{\text{RL}}\left( \lambda-\lambda_{0} \right)\text{d}\lambda={L_{\mathrm{RL}}[D}_{\text{RL},0}\Delta\lambda+S_{\mathrm{RL}}\int_{\lambda_{i}}^{\lambda_{i+1}} \left( \lambda-\lambda_{0} \right) \text{d}\lambda]$$

(S5)

The remaining integral is

$$\int_{\lambda_{i}}^{\lambda_{i+1}} \left( \lambda-\lambda_{0} \right) \text{d}\lambda=\frac{1}{2}\left[ \left( \lambda_{i}+\Delta\lambda-\lambda_{0} \right)^{2}-\left( \lambda_{i}-\lambda_{0} \right)^{2} \right]$$

(S6)

Expanding the square difference,

$$\left( \lambda_{i}+\Delta\lambda-\lambda_{0} \right)^{2}-\left( \lambda_{i}-\lambda_{0} \right)^{2} =2\left( \lambda_{i}-\lambda_{0} \right)\Delta\lambda+\left( \Delta\lambda\right)^{2}$$

(S7)

Inserting Eq. (S7) into Eq. (S6), we obtain

$$\int_{\lambda_{i}}^{\lambda_{i+1}} \left( \lambda-\lambda_{0} \right) \text{d}\lambda= \left( \lambda_{i}-\lambda_{0} \right) \Delta\lambda+\frac{1}{2}\left( \Delta\lambda\right)^{2}$$

(S8)

Combining Eq. (S5) and (S8), the incremental RL dispersion-induced delay between tap $i$ and tap $i+1$ becomes

$$\Delta\tau_{\text{RL,disp}}\left( \lambda_{i} \right) =L_{\text{RL}}[D_{\text{RL},0} \Delta\lambda+S_{\text{RL}}\left( \lambda_{i}-\lambda_{0} \right) \Delta\lambda++\frac{1}{2}S_{\text{RL}}\left( \Delta\lambda\right)^{2}]$$

(S9)

In the special case where $S_{\mathrm{RL}}=0$, Eq. (S9) reduces to Eq. (S1).

2. **Simulation on the effect of dispersion slope**

Although the dispersion slope contributes two terms in Eq. (S9), its impact on the overall filter response is negligible under realistic conditions. To evaluate this, we simulate the recirculating loop using a dispersion-shifted fiber (DSF), where the chromatic dispersion at the center wavelength (1550 nm), $D_{\mathrm{RL},0}$, is set to 0, and the dispersion slope $S_{\mathrm{RL}}$ is swept from 0 to 1 ps (km·nm^2^)^-1^. All other parameters are identical to those used in the main manuscript (e.g., 28 nm ASE bandwidth, 10 km OEO loop). The simulated transfer functions, zoomed to a 1 MHz span to highlight the Q-factor enhancement, are shown in Supplementary Fig. S2. For values representative of practical fibers ($S_{\mathrm{RL}}=0.06$ ps (km·nm^2^)^-1^ and even $S_{\mathrm{RL}}=0.1$ ps (km·nm^2^)^-1^, as given in ITU-T Recommendation G.652), the filter response is nearly indistinguishable from the idealized case with $S_{\mathrm{RL}}=0$, confirming that the RL dispersion slope has a negligible effect. Only when $S_{\mathrm{RL}}$is increased to an unrealistically large value ($S_{\mathrm{RL}}=1$ ps (km·nm^2^)^-1^) do we observe a noticeable reduction in the effective Q-factor. Nevertheless, even in this extreme case, the extinction ratio remains above 2 dB, which is still significantly higher than that achieved with standard single-mode fiber (SMF, $\sim0.4$dB). This further supports our conclusion that the dispersion slope of the recirculating loop does not materially limit system performance. This behavior can be understood as follows. What ultimately matters is that the RL-induced dispersion delay remains as small as possible. The dispersion slope $S_{\mathrm{RL}}$ is typically more than two orders of magnitude smaller than the chromatic dispersion $D_{\mathrm{RL}}$. In Eq. (S9), the second term scales with $(\lambda_{i}-\lambda_{0})$, which is on the order of 10 nm even for a 30 nm ASE bandwidth, so its total contribution to the delay remains much smaller than that of the first ( $D_{\mathrm{RL},0}$) term. The third term scales with $(\Delta\lambda)^{2}$ and is therefore even less significant.

Fig. S2. Simulated effect of the RL’s dispersion slope on the filter response.

We also simulated the filter response using non-zero dispersion-shifted fiber (NZ-DSF), with the dispersion set to 4 ps (km·nm)^-1^ and the dispersion slope set to 0.06 ps (km·nm^2^)^-1^, as given in ITU-T Recommendation G.655. The result is shown in Supplementary Fig. S3. Although the dispersion is relatively small, it still substantially degrades the Q-factor enhancement: the narrow passbands become noticeably flatter, and the extinction ratio is reduced to about 2 dB. Therefore, NZ-DSF is not recommended when true near-zero-dispersion fiber (DSF) is available.

Fig. S3. Simulated filter response using NZ-DSF.

**Supplementary Note 3 | The impact of ASE bandwidth on OEO performance**

In our initial experiment, we chose an ASE bandwidth of 28 nm. This value was not optimized — it was simply a reasonable starting point. However, because the 10 km SMF in the main loop exhibits third-order dispersion, using an excessively wide ASE bandwidth can introduce significant amplitude distortion in the resulting microwave photonic filter. This motivates a more systematic study of how the ASE bandwidth affects OEO performance, so that an appropriate bandwidth can be selected rather than just using the maximum available span.

To isolate this effect, we fixed the optical power at the photodetector to –5 dBm and varied the ASE bandwidth from 10 nm to 15 nm, 20 nm, and 25 nm. We first measured the open-loop filter response, as shown in Supplementary Fig. S4. When the ASE bandwidth increases from 10 nm to 15 nm, the filter bandwidth narrows while the peak amplitude remains essentially unchanged — indicating that third-order dispersion is still negligible over 15 nm. When the bandwidth is further increased to 20 nm, the filter amplitude drops by 1.8 dB. At 25 nm, the drop reaches 3.1 dB, and the passband becomes noticeably broader. Both the amplitude reduction and the spectral broadening at 20–25 nm are attributable to third-order dispersion in the 10 km fiber.

Fig. S4. Measured filter response under different ASE bandwidth

We then examined how this amplitude degradation and passband broadening impact the OEO performance. Specifically, we measured the RF output power for different ASE bandwidths, as shown in Supplementary Fig. S5. For 10 nm and 15 nm bandwidths, the RF output power is essentially identical, consistent with the fact that their filter responses have similar amplitudes. When the ASE bandwidth is increased to 25 nm, the RF output power drops by approximately 1.8 dB, which correlates with the reduced filter amplitude observed in Supplementary Fig. S4.

Fig. S5. Measured RF output power under different ASE bandwidth

Then, we investigate the phase noise performance, from which we can also observe the sidemode suppression ratio. Supplementary Fig. S6 shows the phase noise for the cases using different ASE bandwidths. As can be seen, all the traces are overlapped, which means that the amplitude degradation and bandwidth broadening effect does not harm the sidemode suppression ratio and phase noise.

Fig. S6. Measured phase noise performance under different ASE bandwidth

Overall, the ASE bandwidth has only a minor impact on the OEO performance. Using a slightly narrower ASE bandwidth can provide a modest increase in RF output power (on the order of 1.8 dB), but it does not materially affect phase noise or sidemode suppression. Therefore, in practice, the exact choice of ASE bandwidth is not a critical design parameter and does not require extensive optimization.

**Supplementary Note 4 | The impact of RLs on frequency stability**

The frequency stability reported in the main manuscript is 0.72 kHz over 5 minutes. This value is approximately three times larger than in our previous work, where a similar architecture was used but without the cascaded RLs. This raises the question of whether the reduced stability originates from the added RLs.

To investigate this, we performed an experiment in which the RLs were removed one by one to determine whether eliminating RLs improves frequency stability. The results are summarized in Supplementary Fig. S7. With all RLs present, the measured frequency drift was approximately 1.1 kHz. Notably, this value differs from the 0.72 kHz drift reported in the main experiment. This discrepancy reflects the fact that the measured frequency stability is highly sensitive to ambient temperature fluctuations, which are not well controlled in our laboratory. As a result, the absolute drift value can vary from measurement to measurement. We then removed the 20 m RL, after which the measured drift narrowed to 0.2 kHz. At first glance this might suggest that the 20 m RL degrades stability. However, after subsequently removing the 200 m RL, the drift returned to approximately 1.1 kHz. This indicates that the apparent improvement to 0.2 kHz was not caused by removing the 20 m RL, but rather by measuring during a period when the instantaneous drift rate happened to be particularly low. We consistently observe that the drift rate is not constant over time: at some times the oscillation frequency drifts quickly, and at other times it drifts slowly. This behavior correlates with the varying temperature dynamics in the laboratory environment. Finally, after removing the 1 km RL, the frequency drift remained on the order of 1 kHz. This confirms that removing RLs does not systematically improve frequency stability; conversely, adding RLs does not inherently degrade it. The dominant factor is environmental temperature fluctuation, not the presence of the cascaded RLs.

Fig. S7. Measured frequency stability under different configurations
